# Genetic diversity and population structure of an important wild berry crop

**DOI:** 10.1093/aobpla/plv117

**Published:** 2015-10-19

**Authors:** Laura Zoratti, Luisa Palmieri, Laura Jaakola, Hely Häggman

**Affiliations:** 1 Department of Genetics and Physiology, University of Oulu, PO Box 3000, FI-90014 Oulu, Finland; 2 Department of Food Quality and Nutrition, Research and Innovation Center, Fondazione Edmund Mach, Via E. Mach, 1-38010 San Michele all'Adige (TN), Italy; 3 Department of Arctic and Marine Biology, UiT The Arctic University of Norway, Climate Laboratory, 9037 Tromsø, Norway; 4 Norwegian Institute of Bioeconomy Research, NIBIO Holt, PO Box 115, 1431 Ås, Norway

**Keywords:** Bilberry, genetic diversity, germplasm, ISSR, population structure, *Vaccinium myrtillus*

## Abstract

The success of plant breeding in the coming years will be associated with access to new sources of variation, which will include landraces and wild relatives of crop species. In order to access the reservoir of favourable alleles within wild germplasm, knowledge about the genetic diversity and the population structure of wild species is needed. Bilberry (*Vaccinium myrtillus*) is one of the most important wild crops growing in the forests of Northern European countries, noted for its nutritional properties and its beneficial effects on human health. Assessment of the genetic diversity of wild bilberry germplasm is needed for efforts such as *in situ* conservation, on-farm management and development of plant breeding programmes. However, to date, only a few local (small-scale) genetic studies of this species have been performed. We therefore conducted a study of genetic variability within 32 individual samples collected from different locations in Iceland, Norway, Sweden, Finland and Germany, and analysed genetic diversity among geographic groups. Four selected inter-simple sequence repeat primers allowed the amplification of 127 polymorphic loci which, based on analysis of variance, made it possible to identify 85 % of the genetic diversity within studied bilberry populations, being in agreement with the mixed-mating system of bilberry. Significant correlations were obtained between geographic and genetic distances for the entire set of samples. The analyses also highlighted the presence of a north–south genetic gradient, which is in accordance with recent findings on phenotypic traits of bilberry.

## Abstract (precis)

Bilberry (*Vaccinium myrtillus L.*) fruits are perceived by consumers to be a health-promoting food, conferred by their high content of phenolic compounds and carotenoids. The health properties of these molecules have raised commercial interest in these berries globally, and caught the attention of breeders. Bilberry is a wild crop and represents an important source of genetic variability for blueberry breeding. Our study presents information related to the genetic diversity and structure of bilberry populations derived from Northern Europe, providing new knowledge for the development of genetic studies and for breeding purposes.

## Introduction

The success of plant breeding over the past century has been associated with a narrowing of the available genetic diversity within elite germplasm of species. New sources of variation include landraces and wild relatives of crop species, and although exploiting wild relatives as a source of novel alleles is challenging, it has provided notable successes in crop improvement (Tester and Langridge 2010). Most crop geneticists agree that the enrichment of the cultivated gene pool will be necessary to meet the challenges that lie ahead associated with global environmental changes (Feuillet *et al.* 2008). However, many advances are still needed to access the extensive reservoir of favourable alleles within wild germplasm. These include increasing our understanding of the molecular basis for key traits and expanding existing phenotyping and genotyping of germplasm collections (Feuillet *et al.* 2008). Therefore, knowledge of the genetic diversity and the population structure of wild species is crucial for their management as well as conservation (Burdon and Wilcox 2007; Zhao *et al.* 2014).

*Vaccinium* is a genus of ∼450 plant species in the family Ericaceae that are widely distributed in the Northern Hemisphere and also in the mountains of tropical Asia and Central and South America (Song and Hancock 2011). The species within this genus present different levels of ploidy (2*x*, 4*x* and 6*x*; *x* = 12), which results in evident morphological differences. Regarding domestication and commercial fruit crop production, the most important species are *V. corymbosum* (highbush blueberry), *V. virgatum* (rabbit-eye blueberry), *V. angustifolium* (lowbush blueberry), *V. macrocarpon* (cranberry) and *V. vitis-idaea* (lingonberry). The genus also contains the wild *V. myrtillus* (bilberry) and a number of other currently non-cultivated *Vaccinium* species that show great potential as new berry crops (Song and Hancock 2011). Bilberry belongs to the section *Myrtillus*, and it is a diploid species (2*n* = 2*x* = 24; Song and Hancock 2011). The plant is a deciduous woody dwarf shrub, and it grows typically in pine and spruce heath forests and old peat bogs in Europe, North America, Greenland and northern parts of Asia, including Japan and Greenland (Nestby *et al.* 2011). Bilberry reproduces clonally through rhizomes and also sexually, with an outcrossing rate ranging from 0.66 to 0.75, and it is therefore considered to belong to the group of mixed-mating species (Jacquemart 1993).

Bilberry is an important wild fruit crop, especially in Northern and Eastern European countries, where the berries are picked from the wild and are either sold on the fresh market or frozen for use in food industries to make jams, juices and flavourings. The fruits are also important to the pharmaceutical industry, as they are naturally rich in polyphenols and other antioxidant compounds, which have potential beneficial effects on human health. These berries contain great amounts of flavonoids, in particular anthocyanins, which can reach up to 500 mg/100 g fresh weight (Lätti *et al.* 2008); they also produce carotenoids (Bunea *et al.* 2011) and lower amounts of ascorbic acid (Cocetta *et al.* 2012).

There is an increasing demand for these berries due to their high nutritional value (Martinussen *et al.* 2009), although they are still poorly exploited from a commercial point of view. Despite the fact that the average wild berry yield in Scandinavia has been estimated to be approximately 1 billion kg year^−1^, only ∼5–10 % of the annual crop is utilized for private or commercial consumption (Paassilta *et al.* 2009). Nestby *et al.* (2011) underlined the need for developing an improved production system, in which high yields of good-quality bilberries are produced at manageable costs. So far, cultivation of the species has been very limited and the berries used for commercial purposes are mainly harvested from forests. Therefore, development of forest management systems is considered a good option to achieve this purpose (Nestby *et al.* 2011). Forest management systems will initially require efforts to identify areas in which plants produce high yields and high-quality bilberries. Plants with high-quality characteristics can be identified by phytochemical content or phenotypic traits of interest (e.g. plant productivity, fruit antioxidant content and fruit shelf life) and by genotypic-based methods, whereby the detected molecular polymorphisms are correlated to phenotypic traits. The genotypic-based methods are generally effective, they only need a small amount of DNA (Tanya *et al.* 2011) and they are not affected by environmental factors or developmental stages of the plants. Inter-simple sequence repeats (ISSRs) have shown to be good markers for assessing the genetic diversity of wild *Vaccinium* species from wide geographical areas of collection, in particular, lingonberry (*V. vitis-idaea*; Debnath 2007) and lowbush blueberry (*V. angustifolium*; Debnath 2009). Moreover, ISSR markers were able to detect more polymorphisms than random amplified polimorphic DNA in the same species (Debnath 2009). Therefore, ISSR markers were chosen for our study where the aims were to (i) test the applicability of ISSR markers on bilberry; (ii) determine genetic relationships and diversity among bilberry populations derived from biomes in Northern Europe and (iii) find markers to be used in conservation and management of bilberry in forests of Northern Europe.

## Methods

### Study sites and sampling

**Figure 1.**
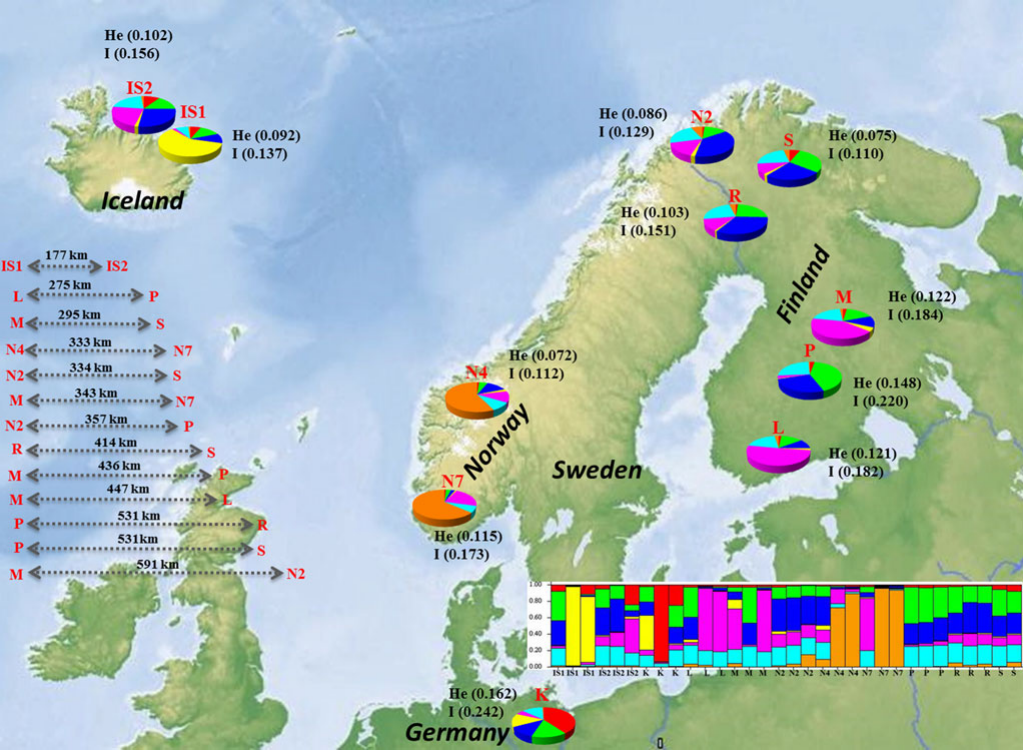
Map of sampling sites in Northern Europe, including ID (according to Table 1). The pie chart represents rather average coefficients of membership resulting from the genetic structure analysis (best fit model, *K* = 7). Each colour represents a different gene pool. The barplot represents each accession as a single vertical bar broken into *K* colour segments, with lengths proportional to the estimate probability of membership in each inferred cluster. Spatial autocorrelation analysis results, and geographical distances of correlated populations, are reported on the left of the figure (ID, grey arrows and geographical distance in kilometres).

Thirty-two individual bilberry samples derived from different seeds collected at different latitudes in several Nordic countries were included in this study (Table 1, Fig. 1): Iceland (IS1, IS2), Norway (N2, N4, N7), Sweden (R), Finland (S, P, M, L) and Germany (K). The plants were established in 2003 from bilberry seeds harvested from a pool of ripe berries collected in an area of 10 × 10 m and micropropagated *in vitro* (Jaakola *et al.* 2001) at the Botanical Gardens of the University of Oulu (Finland). Plantlets were grown in growth rooms under controlled conditions (+22 °C under 16 h photoperiod, white fluorescent Osram 18 W, 1.8 W m^−2^).

**Table 1.** Provenances of bilberry genotypes and genetic diversity parameters based on ISSR markers. Number of samples analysed (*N*), number of different alleles (Na), number of effective alleles (Ne), number of private bands (Np), percentage of polymorphic loci (P %), expected heterozygosity (He) and Shannon's Information index (*I*).

Provenance	ID	Country	Latitude (°N)	Longitude (°E)	Altitude (m above sea level)	Genotype ID	*N*	Na	Ne	Np	P %	He	*I*
Kleifarveugr	IS1	Iceland	66°07′	−18°38′	178	IS1_a, IS1_3, IS1_4	3.000	0.646	1.158	2	24.40	0.092	0.137
Strandavegur	IS2	Iceland	65°47′	−21°22′	10	IS2_a, IS2_1, IS2_5	3.000	0.803	1.161	0	29.92	0.102	0.156
Storfjord	N2	Norway	69°23′	20°16′	3	N2_2, N2_5, N2_6	3.000	0.661	1.142	0	23.62	0.086	0.129
Trondelag	N4	Norway	63°32′	10°53′	420	N4, N4_3, N4_5	3.000	0.614	1.111	0	22.05	0.072	0.112
Storgata	N7	Norway	60°54′	10°44′	173	N7. N7_5, N7_6	3.000	0.772	1.193	0	31.50	0.115	0.173
Kvikkjokk	R	Sweden	66°57′	17°43′	327	R1, R2, R3	3.000	0.693	1.182	2	25.98	0.103	0.151
Sodankylä	S	Finland	67°25′	26°35′	189	S1, S3	2.000	0.630	1.128	2	18.11	0.075	0.110
Muhos	M	Finland	64°48′	25°59′	39	M, M1, M5	3.000	0.835	1.198	2	34.65	0.122	0.184
Parkano	P	Finland	62°02′	23°02′	117	P, P_1, P_10	3.000	0.961	1.251	6	39.37	0.148	0.220
Lapinjärvi	L	Finland	60°37′	26°11′	21	L2, L3, L6	3.000	0.803	1.199	2	33.86	0.121	0.182
Kiel	K	Germany	54°20′	10°08′	14	K2, K6, K10	3.000	0.945	1.276	2	43.31	0.162	0.242

### Genotyping

Genomic DNA was isolated from shoot tips of actively growing *in vitro*-cultured bilberry shoot tips, using the EZNA™ SP Plant DNA Mini Kit (Omega Bio-tek Inc., Norcross, GA, USA) following the manufacturer's instructions. The concentration of DNA was estimated with the NanoDrop N-1000 spectrophotometer (NanoDrop Technologies, Thermo Scientific, Wilmington, DE, USA) at 260 nm. Fifteen primers representing di-, tri-, tetra- and pentamer repeats, previously used to characterize other *Vaccinium* species (Debnath 2007), were considered for the study. Of these, UBC-825, UBC-857, UBC-873 and UBC-881, which gave clear banding patterns, were used for the final study (Table 2). Polymerase chain reaction (PCR) was performed in a final volume of 25 µL including 10 ng of DNA template, 1× Optimized DyNAzyme™ buffer (10 mM Tris–HCl pH 8.3, 1.5 mM MgCl_2_, 50 mM KCl, 0.1 % Triton X-100; Finnzyme, Espoo, Finland), 0.3 µM of each primer, 200 μM dNTPs, 0.8 U of DyNAzyme™ II DNA Polymerase (Finnzyme). The thermal profile consisted of 10 min at 94 °C, followed by 31 cycles of 1 min at 94 °C, 1 min at 46.5 °C and 2 min at 72 °C and a final extension at 72 °C for 10 min. The PCR reaction was purified using sodium acetate–ethanol DNA precipitation. One microlitre of the purified sample was analysed on a capillary electrophoresis system, Agilent 2100 Bioanalyzer with DNA7500 kit (Agilent Technologies, Santa Clara, CA, USA) according to the manufacturer's instructions. Each primer–clone sample combination was repeated at least two times and only replicated bands were included in the analyses. Fragments of similar size across individuals were assumed to be homologous.

**Table 2.** Molecular ISSR primers used for bilberry genotypes' discrimination. Y = (C or T) in ISSR primer sequences; repeat motif and the data on DNA profile and polymorphism generated in 32 bilberry samples; total number of bands (NB), number of polymorphic bands (NPB), proportion of polymorphic bands (PPB), rank of molecular weights (RW); resolving power (Rp).

Primer name	Sequence	NB	NPB	PPB (%)	RW (bp)	Rp
UBC-825	(AC)_8_T	33	31	93.9	310–2100	8.625
UBC-857	(AC)_8_YG	37	37	100	80–6600	13.87
UBC-873	(GACA)_4_	25	25	100	80–2600	9.18
UBC-881	(GGGTG)_3_	32	32	100	60–1400	14.31

### Data analysis

#### Genetic diversity

The amplification product sizes were scored using 2100 Expert Software (Agilent Technologies). The results were transformed into a binomial matrix as present (1) or absent (0) for each marker. Since the ISSR marker is dominant, we assumed that each band represented the single bi-allelic locus (Debnath 2007). Band patterns were analysed in order to determine the level of polymorphism (total number of bands), number of polymorphic bands, proportion of polymorphic bands and the resolving power (Rp) detected for each primer. Resolving power was calculated according to Prevost and Wilkinson (1999); this measure is based on the distribution of alleles among the genotypes and it estimates the discrimination capacity of each primer. Thus, the resolving power of a primer is defined as Rp = ∑Ib, where Ib (band informativeness) takes the value of 1 − [2 × |0.5 − *p*|] and *p* is the ratio of genotypes sharing the band. Moreover, the binomial matrix was used to produce the input matrix following GenAlex version 6.1 software manual instructions (Peakall and Smouse 2006) and analysed using the same software to estimate genetic diversity parameters, i.e. number of different bands, number of different bands with frequency ≥5 %, number of private bands, number of locally common bands frequency ≥5 % found in ≤25 and ≤50 % of populations, mean of expected heterozygosity (He) and the Shannon's Information Index (*I*) calculated as *I* = −1 × (*p* × ln(*p*) + *q* × ln(*q*)), where *p* and *q* are the estimated allele frequencies.

Analysis of molecular variance (AMOVA) was used to partition the total genetic variance into ‘within-populations' or ‘among-populations’ levels. The software GenAlex was used to generate a matrix of pairwise genetic distances between individuals and to calculate the following variance components: degrees of freedom, sum of squares, mean sum of squares, estimated variance and conversion of estimated variances to percentage of total variance. The number of permutations for significance testing was set at 9999. Canonical correspondence analysis (CCA) was done using Past software v. 2.17c (Hammer *et al.* 2001), to determine the relative importance of geographical factors in the spatial organization of genetic diversity among genotypes. This analysis, originally designed for relating species composition to different predictive variables (Ter Braak 1986), has been successfully used to describe the relationship between environmental variables and genetic composition (Angers *et al.* 1999; Girard and Angers 2006; Dell'Acqua *et al.* 2014). The analysis was performed using a geographical variables/genetic data matrix where longitude, latitude and altitude were used as geographic factors. Here, we consider individuals as sites, and alleles at outlier loci as objects. The first three input file columns contain geographical variables, following the CCA Past software v. 2.17c instructions.

#### Spatial genetic analysis

Spatial analysis was conducted using the genetic spatial autocorrelation (SA) (Smouse and Peakall 1999; Peakall *et al.* 2003) option in GenAlex version 6. The pairwise geographical distance matrices were calculated considering them as the crow flies distances in kilometres and were used with the previously obtained genetic distance matrices to generate an autocorrelation coefficient *r* for each distance class using two different options. The autocorrelation coefficient (*r*) is similar to Moran's *I* (Moran 1950) and ranged from −1 to 1. The significance level was tested by constructing a two-tailed 95 % confidence interval around the null hypothesis of no spatial genetic structure, which is *r* = 0. The autocorrelation analysis was performed using a multiple distance class simulation. Since the distance classes have to be small enough to capture the spatial pattern of interest, while large enough to include an adequate number of pairwise comparisons for statistical testing, we characterized the spatial genetic relationship kilometre intervals from 0 up to 2400. This allowed us to determine the strength of autocorrelation and to what extent the autocorrelation decays with increasing distance. To test the null hypothesis of no spatial structure, confidence limits were calculated using permutation and bootstrapping (999 interactions).

Directional autocorrelation analysis was carried out by testing for the direction of maximum genetic correlation using the bearing procedure implemented in PASSAGE version 2 (Rosenberg and Anderson 2011). The bearing method analysed the correlation coefficient (*r*) between geographical distance and genetic relatedness under fixed bearing angles (degrees north of due east). For each sector, *r* is calculated from each distance pair weighted by the cosine of its angle with respect to the angle of the centre arc of the respective sector. Geographical locations were imported with associated genetic data. PASSAGE calculates a distance matrix from genetic data and geographical co-ordinates and an angular matrix from geographical co-ordinates. The correlation was calculated for each 10° sector, from 0° to 180°, and the significance was estimated using 999 randomizations.

#### Grouping of bilberry individuals by STRUCTURE and Cluster analyses

The software STRUCTURE 2.3.3 (Pritchard *et al.* 2000; Falush *et al.* 2003), which, by means of iterative algorithms, identifies clusters of related individuals from multi-locus genotypes, was used to examine the genetic structure of populations. Ten independent runs of STRUCTURE were performed for each *K* value from 1 to 11. Each run consisted of a burn-in period of 100 000 steps, followed by 1 000 000 Markov Chain Monte Carlo replicates, assuming an admixture model and correlated allele frequencies. No prior information was used to define the clusters. The most likely *K* was chosen comparing the average estimates of the likelihood of the data, ln(Pr(*X*|*K*)), for each value of *K* (Pritchard *et al.* 2000), as well as calculating the *ad hoc* statistics Δ*K*, based on the rate of change in the ln probability of data between successive *K* values (Evanno *et al.* 2005). Furthermore, the Past 2.17c (Hammer *et al*. 2001) software was used to generate a matrix using the Dice similarity index. This matrix was used to construct a Ward's dendrogram tree.

## Results

### Genetic diversity

**Figure 2.**
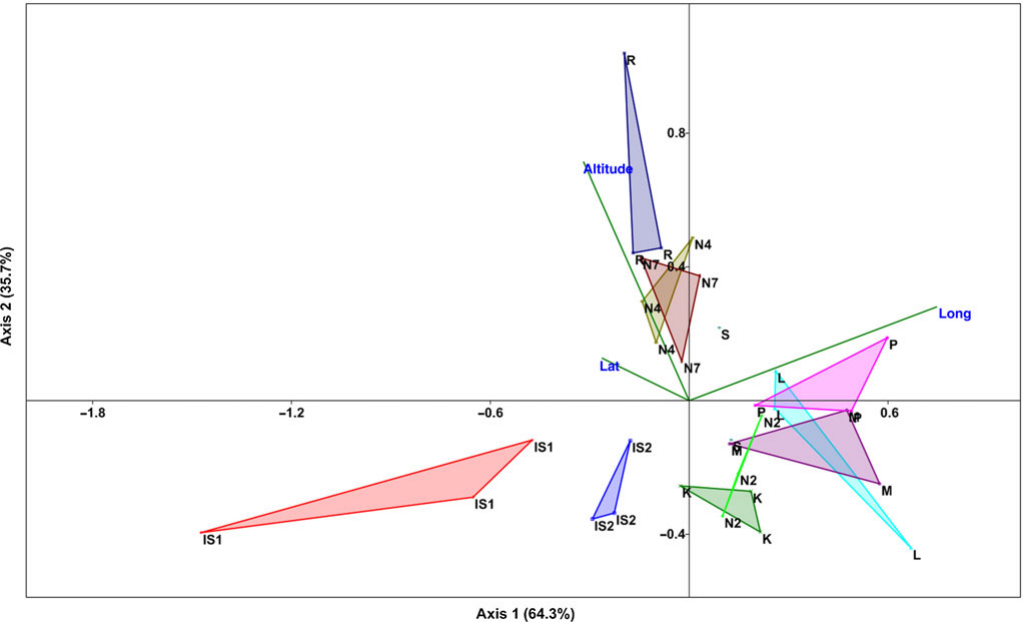
Canonical correspondence analysis ordination biplot representing genotype aggregation and geographical variables (solid arrows). The CCA explained 64.3 and 35.7 % of the variation on the first two axes.

Polymerase chain reaction assays using four primers selected in the initial tests allowed 127 ISSR loci to be amplified from the DNA samples derived from 32 bilberry individuals. The detected loci ranged between 60 and 6600 bp (within the limits of the Agilent DNA7500 kit that allowed the detection of fragments between 50 and 7500 bp). The average number of loci per primer was 31.75, with the highest number of loci (*n* = 37) detected by the UBC-857 primer and the smallest number (*n* = 25) by the UBC-873 primer (Table 2). Of the 127 amplified loci, 126 (99.24 %) were polymorphic. The greatest discrimination power among samples was obtained with primer UBC-881 yielding an Rp value of 14.31, while the lowest Rp value of 8.62 was yielded with primer UBC-825. Except for the primer pair of UBC-825, other primers produced 100 % polymorphic bands (Table 2). The number of bands within populations ranged between 50 and 72, with a number of private bands that ranged between 0 and 6 and a mean expected heterozygosity value that ranged between 0.072 and 0.162. The highest percentage of polymorphic loci (P %) was established in Kiel individuals (43.31 %), while the lowest was observed in S individuals (18.11 %). Moreover, the mean number of different alleles over all loci (Na) for each population ranged between 0.614 and 0.961, the mean number of effective alleles (Ne) ranged between 1.111 and 1.276, and the Shannon's Information Index ranged between 0.110 and 0.242 (Table 1). Analysis of molecular variance indicated that 15 % of the total genetic variance was attributable to among-populations diversity and the rest (85 %) to within-populations diversity. The value of ΦPT (0.190 with a maximum of 0.795) indicated a great level of genetic differentiation among populations. We used a CCA to investigate the possible aggregation or differentiation of analysed genotypes. Results of the CCA (Fig. 2) revealed the strong influence of the geographical position on genotype aggregation (Axis 1 = 63.3 % of variance; Axis 2 = 35.7 %). The correlation biplot, which considers both the direction and the relative length of the vectors, underlines clustering for most of the individuals having the same origins. For instance, the clustering of Finnish individuals (P, L, M and one S sample, Fig. 1) occurred according to longitude, and the clustering of the same Finnish samples with Norwegian individuals derived from the closer latitudes (Fig. 1) was evident. The individuals from Norway (N4 and N7) overlapped and were in close proximity to Swedish individuals probably due to the same influence of all geographical variables on all these samples. IS1, IS2 and K individuals are clustering alone as previously shown, but IS1 and IS2 seem to be strongly influenced by negative longitude values while the *K* individuals seem to be most influenced by lower values of latitude.

### Spatial genetic structure

**Figure 3.**
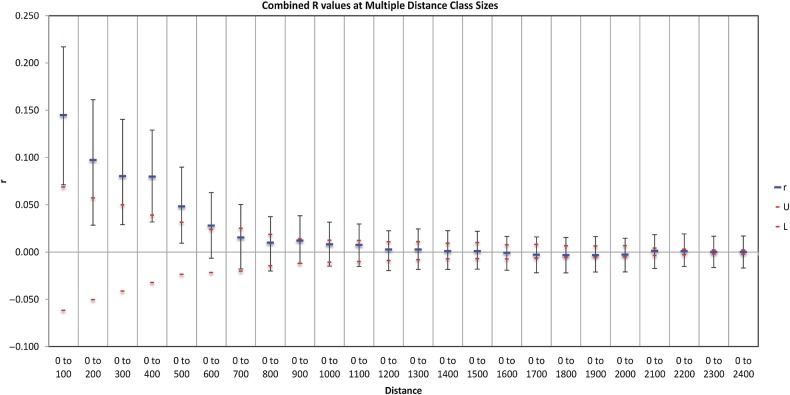
Results of multiple SA analyses for increasing distance class sizes to determine SA. Confidence limits for the *r* values are indicated and were estimated by permutation (999 interactions). Upper (U) and lower (L) confidence limits were generated for the null hypothesis of no SA (*r* = 0) by bootstrap (999 interactions).

In the correlogram that resulted from SA analysis, the *y*-axis average SA coefficient, *r*, has a function of distance class on the *x*-axis. The maximum number of distance classes obtained using the even sample classes options was 52. It is apparent in Fig. 3 that there is a highly significant positive SA (the *r* value falls above or at the 95 % confidence interval, close to a correlation of zero) at distance classes 0–100 evidencing non-random spatial genetic structure within populations. Moreover, the positive correlation decreases until 600 km (*r* = 0.028). Figure 1 reports the IDs and the geographical distances of populations showing positive *r* values. The bearing analysis from PASSAGE indicated the strongest correlation occurred along a north–south axis (*r* = 0.856, *P* = 0.001), while the weakest occurred in the east–west direction (*r* = −0.188, *P* = 0).

### Clustering of bilberry individuals by STRUCTURE analysis

STRUCTURE analysis assigned genotypes into respective groups on the basis of their allele frequencies. According to the user-defined settings, the programme assumed that genotypes are admixed and the allele frequencies are correlated as a consequence of shared ancestry and/or migration. Bayesian cluster, based on an admixture model, presumes that each individual has inherited some proportion of its ancestry from each of the *K* genotypes (Pritchard *et al.* 2000). According to the Evanno's method, the STRUCTURE analysis indicated *K* = 7 as the most likely number of gene pools. These pools represented most geographical groups but with a substantially different proportion of membership (*q*) of each group in each gene pool. Considering the mean value for each geographical group of genotypes (*Q*), six different patterns of genetic makeup were evidenced. The first type of pattern included two Norwegian groups (N4 and N7) out of three with the highest *Q* in gene pool 7 (0.572 and 0.636, respectively). Finnish populations represented two distinct patterns with the highest *Q* value in gene pool 5 for L and M groups (0.508 and 0.420, respectively) and in gene pool 2 for P and S groups (0.411 and 0.295, respectively). The IS1 population had the maximum *Q* value in gene pool 4 (0.59) and the *K* group in gene pool 1. Finally, individuals from Iceland (IS2), Norway (N2) and Sweden (R) had similar *Q* values in gene pools 3, 6 and 7 (Fig. 1).

**Figure 4.**
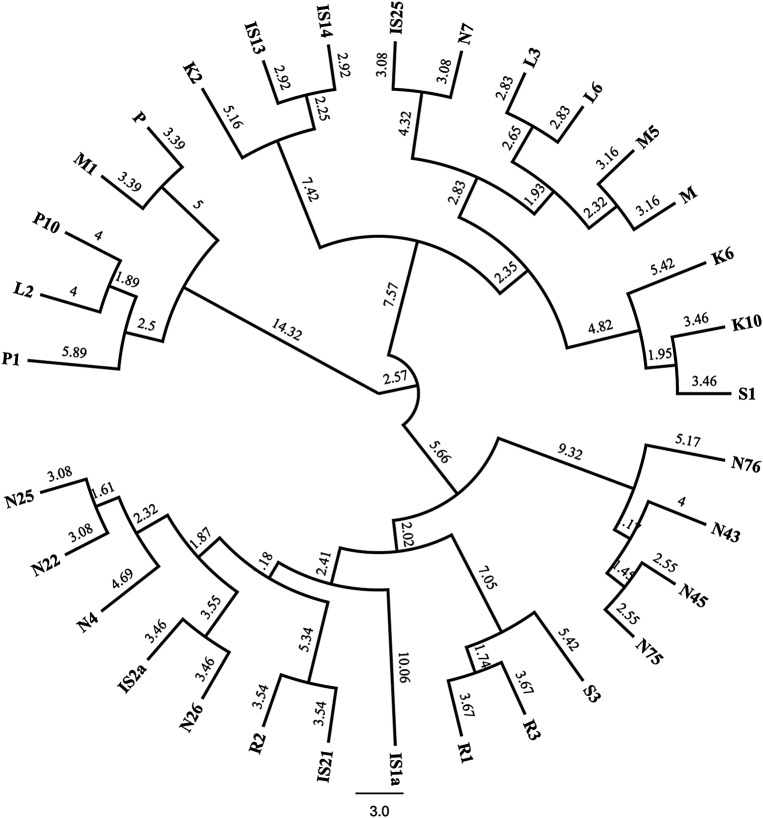
Dendogram of bilberry individuals using Ward's method. Numbers above branches indicate bootstrap values based on 10 000 replicates.

The Dice distances between pairs of populations were calculated based on the 127 analysed bands. The dendrogram was built using Ward's method (Fig. 4) and showed clusters comparable with clusters evidenced from the STRUCTURE analysis, except for P and R genotypes, which clustered alone as previously assessed.

## Discussion

Plant genetic resources are essential for sustainable agriculture and food security. One of the best ways to preserve them is *in situ* management, which becomes particularly important in cases of wild crop species such as bilberry. Molecular markers can provide important information regarding genetic polymorphism and essential knowledge for development and improvement of plant populations; however, to date only a few studies have been carried out on the assessment of bilberry germplasm genetic diversity (Raspé *et al*. 2004; Albert *et al*. 2008).

In our study, ISSR markers were used to evaluate genetic variation among 32 bilberry individuals located in Fennoscandia and Germany. Four primers detected significant genetic variation among the genotypes thanks to their high polymorphism level (99 %). A similar high level of diversity in the same genus (80.4 %) was reported in highbush and rabbit-eye blueberry (Garriga *et al*. 2013). The ability to discriminate bilberry samples varied between different ISSR primers, in agreement with the findings in lingonberry (Debnath 2007) and lowbush blueberry (Debnath 2009).

The high percentage value of genetic diversity within-population obtained from AMOVA can be explained as a natural selection mechanism to reduce fitness costs due to geitonogamous self-pollination in bilberry as previously reported by Albert *et al*. (2008). The same results were also found in other wild *Vaccinium* species such as lingonberry (Persson and Gustavsson 2001; Garkava-Gustavsson *et al*. 2005; Debnath 2007), bog bilberry (*V. uliginosum*; Eidesen *et al*. 2007) and wild lowbush blueberry (Debnath 2009). Moreover, the great genetic differentiation among geographically distant populations, determined from the positive value of ΦPT, is in accordance with the theory that the level of genetic heterogeneity among individuals is greater in species with geographically disjunctive populations than in species with more continuous distributions (Hamrick and Godt 1996; Premoli *et al*. 2001). Therefore, in the present study, we focussed on the genetic diversity of bilberry plants separated by long distances, clones of which are discontinuous and isolated from one another by mountains or seas. The effect of these natural barriers together with common results of different clusters and spatial and population structure analyses provided evidence of a great association between the Finnish and the Norwegian N2 populations on one side, the Norwegian populations N4 and N7 on the other side, and split IS and K populations from all the others. Moreover, the presence of a north–south genetic gradient is in accordance with recent findings on bilberry phenotypic traits (Lätti *et al.* 2008; Åkerström *et al.* 2010; Uleberg *et al.* 2012). These results are also supported by other studies carried out on different species that show how intra-specific genetic variation increases or decreases in relation to the physical distance separating the individuals, and is also showing the influence of the geographic structure of natural populations (Brooks *et al.* 2015). To date, most conservation activities have focussed on the species level; however, also genetic variation at an intra-specific level needs to be considered to avoid loss of diversity derived from severe inbreeding, resulting in lowered fitness and increasing risk of extinction. Moreover, the determination and the conservation of the within-population genetic diversity of one species could increase evolutionary resilience when different and geographically separated environments are connected. When populations are interconnected along climatic and geographical gradients, there is the potential for *in situ* adaptive evolution (Sgrò *et al. 2*011). Furthermore, plant species conserved in key biodiversity areas are an essential genetic source to develop new varieties for future breeding work and to avoid the diversity loss derived from severe inbreeding. Finally, genetic diversity conservation might become crucial in a biotic or abiotic crisis where only very rare genotypes may be resistant to a new disease, pathogen strain or environmental condition. This was further supported by the highest values for He, Shannon Index and number of polymorphic bands.

Further studies with a higher number of markers and samples from the distribution area of bilberry are needed to identify the key biodiversity areas of the species.

## Conclusions

Advances in genotyping techniques combined with more sophisticated statistical methods provide the means by which among- and within-population genetic diversity can be estimated in the absence of any prior specific information. This valuation is necessary to conserve the biodiversity of specific areas. In this perspective, we assessed the intra- and inter-population genetic diversity of 32 individuals collected from different North European countries. We found the presence of significant correlations between geographic and genetic distances, which placed Norwegian, Finnish, Icelandic and German genotypes in separate groups. The present results indicate how key biodiversity areas of the wild *V. myrtillus* species could be individualized as a useful source of biodiversity for future ecological studies and breeding purposes.

## Sources of Funding

The study was funded by the Thule Institute (2013–16) (to H.H.) and by Nordic Innovation Centre—New Nordic Food project ‘Bilberry: Towards functional food markets’ (2007–10) (to H.H. and L.J.).

## Contributions by the Authors

L.Z. performed the DNA analyses and the ISSR data scoring and was involved in writing and editing; L.P. performed all the statistical analyses and was involved in writing and editing; L.J. and H.H. provided contribution to the concept and the design of the work. All authors read and approved the final manuscript.

## Conflict of Interest Statement

None declared.
